# Modernizing Colorectal Cancer Care With Artificial Intelligence: Real-Time Detection, Radiomics, and Digital Pathology

**DOI:** 10.7759/cureus.95178

**Published:** 2025-10-22

**Authors:** Elmoatazbellah Nasr, Zaid Al-Hamid, Mina H Younan, Mohamed Omran, Maan Sarsam, Mohamed Abdellatif

**Affiliations:** 1 General Surgery, Calderdale and Huddersfield NHS Foundation Trust, Huddersfield, GBR; 2 General and Colorectal Surgery, Blackpool Teaching Hospitals NHS Foundation Trust, Blackpool, GBR; 3 General Surgery, Hull University Teaching Hospitals NHS Trust, Hull, GBR; 4 Surgery, University Hospitals Bristol and Weston NHS Foundation Trust, Bristol, GBR; 5 General Surgery, University Hospitals of Leicester NHS Trust, Leicester, GBR

**Keywords:** artificial intelligence (ai), colonoscopy, colorectal cancer (crc), computer-aided detection (cade), deep learning, digital pathology, radiomics

## Abstract

Colorectal cancer (CRC) remains a major global burden, demanding earlier detection and more precise care. Artificial intelligence (AI) is reshaping the CRC pathway by boosting lesion detection, expediting molecular triage, and enabling quantitative, multimodal decision support. Evidence shows computer-aided detection increases adenoma detection and lowers miss rates; digital pathology can infer microsatellite instability from routine slides to prioritize confirmatory testing. AI also strengthens CT/MRI via segmentation, radiomics-based risk stratification, nodal staging, and response prediction, while blood- and genomics-driven models extend noninvasive screening and prognosis. Translating these gains requires high-quality data, external validation, interpretability, workflow integration, and robust governance. Priorities include multicenter prospective studies, lifecycle performance monitoring, and implementation frameworks that ensure usability, equity, and cost-effectiveness, enabling AI to evolve into a dependable infrastructure that improves CRC outcomes.

## Introduction and background

Colorectal cancer (CRC) is a major global health burden, responsible for roughly 10% of all cancer diagnoses and deaths worldwide. Among malignancies, it is estimated to be the third most common and the second leading cause of cancer-related mortality [1,2]. A higher colonic precancerous lesion detection during colonoscopy is associated with lower post-colonoscopy CRC incidence [3-7]. This emphasizes the ongoing necessity of early cancer detection and the timely management of precancerous lesions. Evidence shows that colonoscopy markedly lowers the 10-year risk of CRC compared with no screening, as reported by Bretthauer et al., underscoring its value for early detection and prevention. A single screening colonoscopy reduces CRC risk by 18%, with a risk ratio of 0.82 [8].

The ongoing need for CRC incidence reduction through early polyp detection is highlighted. A meta-analysis of more than 15,000 colonoscopies showed substantial under-detection, with an adenoma miss rate of 26% and a serrated polyp miss rate of 27%, underscoring the need for improved detection methods [9]. Missed colorectal neoplasia represents the predominant mechanism underlying post-colonoscopy CRC, which occurs in approximately 0.6-4% of individuals following screening or surveillance colonoscopy [4-7].

Artificial intelligence (AI) in medicine spans medical imaging, clinical decision support, computational drug discovery, and surgical robotics. With accelerating adoption, oncology is seeing broad benefits. In CRC, recent studies indicate that AI improves early detection and is associated with higher post-treatment five-year survival [10]. Meta-analysis data show clinically meaningful gains with AI assistance, adenoma detection rate rises alongside increases in adenomas per colonoscopy and detection of non-advanced lesions, key for preventing future cancer [11,12]. In this review, we investigated the current state of modern colorectal surgery practice, highlighting the use of AI, radiomics, and digital pathology through the literature.

## Review

Methodology

We undertook a narrative search of the literature. Sources included PubMed/MEDLINE, Embase, Web of Science, Scopus, and the Cochrane Library. Targeted hand-searches covered major gastrointestinal (GI)/endoscopy society statements (e.g., European Society of Gastrointestinal Endoscopy/American Society for Gastrointestinal Endoscopy), radiology/pathology journals, and implementation reports. Search strings combined terms for CRC with “colonoscopy,” “computer-aided detection/CADe,” “radiomics,” “deep learning,” “digital pathology,” “microsatellite instability,” and “H&E.” Reference lists of key articles and recent meta-analyses were snowballed to identify additional high-yield studies. Evidence was synthesized narratively, grouping results by technology class and clinical question; quantitative pooling was not planned.

Scope and Approach

This is a narrative, thematically organized, non-exhaustive review that synthesizes representative, high-signal sources across three pillars of CRC AI: (i) real-time endoscopic computer-aided detection in colonoscopy (CADe), (ii) radiomics/AI-enhanced CT/MRI, and (iii) digital pathology (including microsatellite instability (MSI) inference from hematoxylin and eosin (H&E)). We privilege studies that materially inform practice (e.g., randomized controlled trials (RCTs), multicenter validations, consensus statements) and use supportive examples (e.g., blood-based/omics AI) only where they clarify pathways of care. The aim is to map decisive trends, typical effect sizes, and implementation considerations rather than enumerate every publication.

Pragmatic Selection Principles

When multiple options existed, we preferred higher-level evidence (systematic reviews/meta-analyses, randomized or prospective multicenter trials, large registries) and externally validated models. Single case reports and very small, single-arm series were excluded unless uniquely illustrative of a method or failure mode. We emphasized consensus-backed work (e.g., society position statements) and studies with transparent methods (public code/data, prespecified endpoints, calibration reporting). Recency (≈2020-2025) and clinical salience (adenoma detection rate/adenoma miss rate for CADe; area under the receiver operating characteristic curve/calibration/external validation for pathology and radiomics) guided tie-breaks.

Computer-aided detection in colonoscopy

CADe systems are a leading application of AI in CRC endoscopy, providing real-time assistance during colonoscopy by automatically flagging polyps. These systems include CADe systems such as GI Genius (Medtronic), Fujifilm CAD EYE, EndoScreener (built on SegNet), and platforms leveraging YOLO architectures [11].

**Figure 1:**
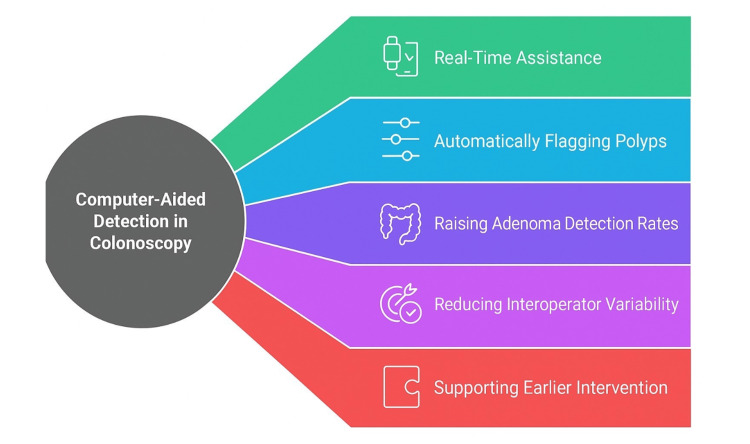
Computer-aided detection in colonoscopy. This figure was created by the authors summarizing the use of computer-aided detection in colonoscopy according to references [11-14].

CADe raises adenoma detection rates, particularly for subtle, diminutive, or flat lesions, thereby reducing dependence on individual operator experience. In doing so, CADe helps curb interoperator variability and supports the standardization of colonoscopy quality metrics. Leveraging AI in this manner provides a practical route to enhance detection consistency and overall procedural quality, making CADe a promising tool to harmonize care and, ultimately, reduce CRC incidence and mortality [13]. CADe consistently improves detection performance, supporting earlier intervention and potentially lowering interval CRC risk (Figure 1) [12-14].

A recent meta-analysis pooling 44 RCTs that compared standard colonoscopy with AI-driven CADe across screening, surveillance, and diagnostic settings found consistent improvements in detection outcomes. CADe increased the adenoma detection rate from 36.7% to 44.7% (risk ratio (RR) = 1.21; 95% confidence interval (CI) = 1.15-1.28) and raised adenomas per colonoscopy from 0.78 to 0.98 (incidence rate difference = 0.22; 95% CI = 0.16-0.28). In tandem colonoscopy designs, CADe significantly reduced the adenoma miss rate from 35.3% to 16.1% (RR = 0.47; 95% CI = 0.36-0.60). Platform-specific effects were also noted: YOLO-based systems increased adenoma detection rates from 22% to 29% (RR = 1.36; 95% CI = 1.14-1.62), GI Genius from 50% to 55% (RR = 1.16; 95% CI = 1.00-1.34), Fujifilm CAD EYE from 43% to 53% (RR = 1.21; 95% CI = 1.10-1.34), and EndoScreener from 26% to 31% (RR = 1.22; 95% CI = 1.11-1.35) [12]. Moreover, a meta-analysis of six tandem RCTs (1,718 patients) showed CADe versus standard white-light colonoscopy reduced adenoma miss rate by 54% and polyp miss rate by 56%, with low heterogeneity (18%). Sensitivity analyses confirmed benefits in screening/surveillance, supporting CADe for accurate CRC screening [15].

Convolutional neural networks enable frame-by-frame inference at clinical frame rates, localizing diminutive and flat lesions without interrupting workflow. Early prospective work demonstrated robust real-time polyp identification on commodity hardware, foreshadowing subsequent randomized trials and regulatory clearances [16].

Deep learning-based artificial intelligence in tumor pathology

In CRC, a major genomic change is MSI, which results from defects in the mismatch-repair pathway and is found in about 5-20% of tumors [17]. MSI prevalence varies by stage, exceeding 20% in stage II CRC but falling below 5% in later stages [18].

Pathologists have long sought morphologic hallmarks of MSI tumors, such as tumor-infiltrating lymphocytes and mucinous architecture, on H&E-stained slides. Yet, these features are difficult to quantify by hand, and interpretations vary between observers. To address these limitations, researchers have developed AU methods that infer MSI status directly from whole-slide images (WSIs) across multiple cancer types [19-21].

Deep-learning models applied to routine H&E WSIs can infer MSI by capturing morphologic signatures of mismatch-repair deficiency. Across multi-institutional cohorts with external validation, these systems achieve clinically useful discrimination and generalize across scanners and populations. Evidence supports their use as a rapid, low-cost pre-screen to enrich formal molecular testing, streamline reflex workflows, and prioritize confirmatory assays, thereby accelerating diagnosis and reducing unnecessary tests while maintaining high negative predictive value in settings and care pathways [22,23].

Kather et al. developed the first automated, end-to-end deep-learning model for MSI/deficient mismatch repair detection in 2019, achieving an impressive area under the curve (AUC) of 0.84 within the TCGA cohort. Following this, further studies utilizing new methodologies have shown even better AUC values, ranging from 0.78 to 0.98 [19].

By introducing WSIs, digital pathology now allows detailed, shareable tissue analysis, propelling AI-driven diagnosis. The result is better cancer identification subtyping and outcome prediction, enhancing tailored treatment [19,24-26]. However, these models are still at their elementary level, with limited data for validation [27]. In 2022, these advances culminated in the first deep-learning biomarker detector, MSIntuit (Owkin; Paris/New York), receiving approval for usual clinical use in Europe [28].

However, many obstacles to AI in digital pathology persist, including scarce expertly labeled datasets; pervasive histologic variability requiring many examples per pattern; diagnostic judgments that rarely fit simple binary categories; gigapixel WSIs that force patching and risk downsampling losses; reliance on weak, single-task models; substantial computational and storage demands; susceptibility to adversarial perturbations and common slide artifacts; and black-box decision-making that limits interpretability, trust, and regulation. Beyond algorithms, deployment realities matter: full automation is neither realistic nor wise; the clinician remains the ultimate evaluator, and adoption depends on usability, clear return on investment, and demonstrated performance [20].

Artificial intelligence for colorectal cancer imaging

Growing evidence indicates that AI could reshape healthcare, especially tasks involving image analysis [29-31]. In colorectal disease, applications already span polyp and adenoma detection, CRC assessment, ulcerative colitis evaluation, and intestinal motility disorders [32-34]. With rapid advances in technology, AI is poised to remain a key driver of progress in colorectal diagnosis and therapy [10].

Radiomics techniques convert medical images into high-dimensional quantitative data, capturing tumor heterogeneity that is not apparent to the naked eye [35]. In CRC, radiomics is rapidly maturing as a tool to refine disease stratification. CT-derived radiomic signatures have differentiated high- from low-grade tumors (AUC = 0.7-0.9), separated stage I-II from stage III-IV disease (AUC = 0.8), and predicted MSI, a marker with therapeutic implications. Crucially, radiomics should complement, not replace, standard clinicopathologic assessment. In a recent RCT, a combined clinical-radiomics model applied to preoperative CT improved MSI prediction over either component alone (AUC = 0.8) [36].

AI-based segmentation systems help delineate colorectal tumors on imaging. Modern deep-learning models provide highly accurate, automated lesion segmentation on MRI and CT, which reduces interobserver variability. In turn, precise tumor contours improve the consistency of volumetric measurements, sharpen local staging, and optimize radiotherapy target definition and planning [37].

Deep learning is increasingly effective for lymph-node assessment on imaging. A recent meta-analysis found that convolutional neural networks reached an AUROC of ~0.92 for detecting nodal metastases on preoperative scans, substantially outperforming radiologists (≈0.68). With continued refinement, such models could markedly enhance preoperative nodal staging accuracy in CRC [38].

AI is increasingly used to forecast therapeutic response. Radiomics and deep-learning models derived from pretreatment MRI can flag rectal cancer patients likely to achieve a pathological complete response after neoadjuvant chemoradiation. Similarly, multimodal radiomic signatures that fuse MRI and PET features have shown strong preoperative accuracy in predicting who will respond to therapy [36].

During post-treatment follow-up, AI-enhanced image analysis supports early identification of recurrence risk. In localized colon cancer, baseline CT radiomics models stratify relapse risk, with five-year relapse AUC ~0.74 [39].

**Figure 2:**
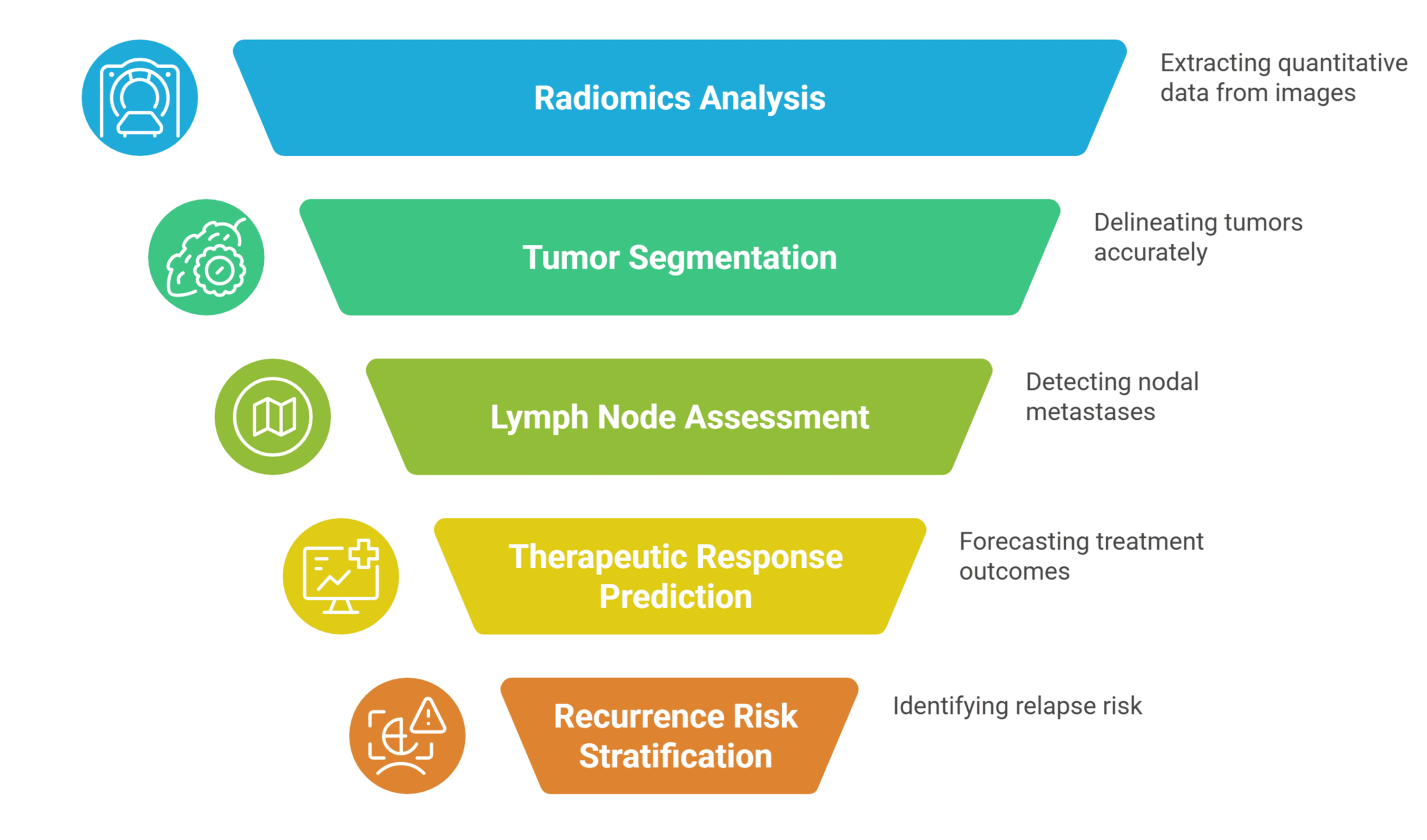
Artificial intelligence (AI)-enhanced colorectal cancer diagnosis. This figure was created by the authors to highlight the main benefits of AI-assisted colorectal cancer diagnosis. Sources: [36-40].

Multi-modal integration and advanced decision-support systems are on the horizon. By combining radiologic features with genomic, pathological, and clinical data, AI can provide comprehensive insights for personalized care. These radiogenomic and multi-omics models promise to refine prognostication and treatment selection in CRC management. Overall, the incorporation of AI into CT and MRI for CRC augments clinical decision-making. Quantitative imaging biomarkers and predictive models derived through AI serve as evidence-based aids for clinicians, improving risk stratification and guiding tailored therapeutic strategies and follow-up plans (Figure 2) [36,40].

Applying artificial intelligence to blood analyses and related diagnostics for colorectal cancer

Blood tests are noninvasive, accurate, and relatively low-cost; boosting their precision could advance early CRC detection. Using blood fluorescence spectroscopy, Soares et al. trained a support vector machine (SVM) model that distinguished CRC from normal tissue with 87% sensitivity and 95% specificity and identified nonmalignant findings with 60% sensitivity and 79% specificity [41]. ColonFlag, a machine-learning tool that leverages demographics and complete blood counts, was evaluated in 17,676 individuals. A positive score doubled the odds of advanced precancerous lesions at 95% specificity, guiding intensified colonoscopy screening [42]. The CellMax (CMx®) platform enriched epithelial circulating tumor cells, showing 100% experimental specificity and 80% clinical sensitivity in a 47-subject cohort, supporting clinical feasibility [42,43]. Beyond cells, AI applied to serum protein biomarkers also enabled noninvasive CRC diagnosis [44].

AI similarly augments genetic testing in CRC. Hu et al. compared three models using gene expression profiles from Union for International Cancer Control II cases, finding S-Kohonen networks classified relapse versus no relapse with 91% accuracy, outperforming back-propagation (66%) and SVM (70%) [45]. Xu et al. later used an SVM pipeline to identify differentially expressed genes, validating a 15-gene panel that stratified high-risk patients and predicted prognosis [46].

Methodological advances continue across epigenetic and mutation targets. Kel et al. introduced a “walking pathway” strategy to discover methylated DNA biomarkers and used AI to interrogate cancer-specific enhancers in CRC [47]. Zhang et al. applied a counter-propagation artificial neural network to near-infrared assays for *BRAF V600E*, achieving 100% sensitivity, 87.5% specificity, and 93.8% accuracy, also discriminating mutation from wild type at lower cost [48].

Limitations and future directions

There are still several difficulties. Large, varied datasets are necessary for AI models to be validated thoroughly and successfully incorporated into standard healthcare workflows. It takes a long time to train these models as they frequently require hundreds or thousands of annotated photos, which may require considerable hand labeling. Issues with model interpretability, data privacy, and regulatory approval further complicate implementation. To guarantee the effective and secure integration of AI in clinical practice, these obstacles must be addressed [27].

Integrating AI into colonoscopy may unintentionally deskill endoscopists, with over-reliance on automation eroding diagnostic skills [49,50]. Evidence includes a study highlighted after AI adoption: adenoma detection rates during non-AI colonoscopies fell from 28.4% to 22.1%, a 6.3% absolute (22.2% relative) reduction, suggesting diminished performance when AI assistance is absent during routine clinical practice [49].

Most AI systems depend on large volumes of high-quality training images that are ideally labeled (annotated). Typically, a physician must manually outline regions of interest (e.g., anomalies or malignancy) across images, with expert annotation preferred. This process is time-intensive and costly, creating a bottleneck for development. Crowdsourcing can be faster and cheaper, but may introduce label noise. For physicians, bulk annotation can be tedious and is harder with blurry/low-resolution images, slow networks, or ambiguous features. Active learning can reduce the burden. Currently, only a few publicly available labeled datasets exist [20].

Future Directions

AI has entered a new phase marked by rapid progress in domain-specific, professional use cases. Many systems remain pre-deployment, but advances in computing power, data availability, and model architectures, along with evolving regulation and validation frameworks, make their routine adoption plausible within the next 10-15 years [51].

AI-based models are rapidly emerging across numerous medical specialties-including radiology, dermatology, ophthalmology, and pathology, with early evaluations consistently report promising performance, suggesting meaningful potential to enhance diagnostic accuracy, workflow efficiency, and clinical decision-making in routine practice [24,52-54].

Within pathology, early AI efforts largely mirrored human tasks (segmentation, classification, and grading) to reduce observer variability, but future work must confront data and deployment realities by mitigate overfitting, class imbalance, and selection bias; move beyond TCGA-centric training by curating large, ethnically diverse, expert-reviewed WSI datasets; and mandate robust external, multi-institution validation. Clinically, prospective, and, where feasible, randomized, studies should establish utility, safety, and workflow impact. Because many stage IV CRC cases provide only endoscopic biopsies, models require optimization and validation on small tissue samples to guide immunotherapy selection. Differentiating Lynch syndrome from sporadic MSI-H remains an unmet need. Finally, the “black-box” nature of deep models necessitates standardized reporting, explainability audits, and benchmarking against pathology quality standards to ensure trustworthy adoption in routine practice [27].

Additionally, future research should prioritize multicenter, adequately powered trials with long-term follow-up (≥10 years) to assess patient-important outcomes after CADe-assisted colonoscopy, CRC incidence, stage at diagnosis, post-colonoscopy CRC rates, and disease-specific mortality, alongside cost-effectiveness, real-world implementation, and subgroup effects (e.g., lesion morphology, operator experience) [11].

## Conclusions

AI is reshaping CRC care from screening to survivorship by boosting detection, refining diagnosis, and guiding treatment. In endoscopy, real-time computer-aided detection raises adenoma detection and lowers miss rates, reducing post-colonoscopy CRC. Digital pathology infers traits such as MSI from H&E slides, expediting triage. Radiology gains via segmentation and radiomics that stratify risk and predict nodal status and therapy response, while blood, genomic, and epigenetic models broaden noninvasive screening. It is also important to note that AI must be embedded in multidisciplinary workflows that individualize surveillance intervals, neoadjuvant strategies, surgical planning, and adjuvant therapy. Progress hinges on rigorous external validation, bias mitigation, interpretable deployment, and privacy-aware, regulated, usable systems. Priorities include multicenter prospective trials with patient-important endpoints, continuous performance monitoring, and governance that ensures accountability, cost-effectiveness, clinician competence, and measurable improvements in CRC outcomes and value of care.
